# Reverse design of broadband sound absorption structure based on deep learning method

**DOI:** 10.1038/s41598-025-86077-w

**Published:** 2025-01-14

**Authors:** Yihong Zhou, Lifeng Ma, Xi Kang, Zhiyuan Zhu

**Affiliations:** School of Mechanical and Automotive Engineering, Shanghai University of Engineering Science, Shanghai, 201620 China

**Keywords:** Sound-absorbing materials, Deep learning, Neural networks, Reverse design, Lightweight design, Target optimization, Engineering, Materials science

## Abstract

This research presents a method based on deep learning for the reverse design of sound-absorbing structures. Traditional methods require time-consuming individual numerical simulations followed by cumbersome calculations, whereas the deep learning design method significantly simplifies the design process, achieving efficient and rapid design objectives. By utilizing deep neural networks, a mapping relationship between structural parameters and the sound absorption coefficient curve is established. The forward network predicts the sound absorption coefficient curve, while the reverse network enables the on-demand design of structural parameters for broadband high sound absorption. During the design process, a mean squared error (MSE) below 0.0001 is achieved. The accuracy of the proposed design method is validated through examples. The results demonstrate that the trained deep learning neural network could effectively replace the complex physical mechanisms between structural parameters and sound absorption coefficient curves. This deep learning design method could also be extended to other types of metamaterial reverse designs, significantly enhancing the efficiency of complex metamaterial designs. Lightweight design is crucial for energy saving and emission reduction. With the total mass and average sound absorption coefficient of sound-absorbing materials as targets, the NSGA-II algorithm has been used for multi-objective optimization design. The optimized average sound absorption coefficient increased by 4.84%, and the total material mass was reduced by 18.98%.

## Introduction

Noise has a significant impact on our daily lives and has long been a concern for scientists and engineers in the field of acoustics, covering various aspects such as environmental, industrial, and traffic noise^1^. Noise control within vehicle cabins has been a prominent topic in the automotive industry^2^. In recent years, NVH performance has gradually become a crucial criterion for assessing vehicle quality and product positioning^3^. The use of acoustic packages is an effective method to reduce the interior vehicle noise. Therefore, the design of automotive acoustic packages has now become an important research topic in the automotive industry^4^.

Generally, acoustic absorbing materials could be classified into porous absorbing materials and resonant absorbing structures. Maa^5,6^ proposed the classic microperforated panel (MPP) and introduced an acoustic structure composed of two MPP layers with different structural parameters. It has been demonstrated as an effective and simple method to reduce noise within the mid to low-frequency range. Bucciarelli et al.^7^ presented one multi-layer MPP absorbing material with high sound absorption coefficient and broad low-frequency absorption characteristics. Sound-absorbing cotton (SAC) is a traditional porous absorbing material mainly used for absorbing mid to high-frequency noise^8–10^. This research work proposes enhancing noise absorption performance across the full frequency range through a combination of composite microperforated panels and porous materials.

Recently, deep learning technology is rapidly advancing, offering new strategies for engineering applications and emerging as a promising method for solving complex problems^11^. They intelligently express data features through network models composed of multiple hidden layers using the backpropagation algorithm^12^. Deep learning methods have shown unique advantages in optimizing the design of photonic crystals^13^. Gao et al.^14^ predict the on-demand design of Helmholtz resonators using deep autoencoder networks. Wang et al.^15^ perform on-demand reverse design of acoustic metamaterials based on probabilistic generative networks. Ma et al.^16^ achieve inverse design of broadband stealth absorbers using convolutional autoencoder networks and inverse design networks. Song et al.^17^ design laminated soundproof metamaterials using deep learning methods. Yang et al.^18^ predict the sound absorption coefficient of ultra-porous materials using convolutional neural networks. K. Mahesh^19^ proposes an algorithm to perform the inverse design of a low-frequency acoustic absorber using a deep convolutional autoencoder network. Mahesh^20^ proposes a deep neural network based inverse prediction mechanism is proposed to geometrically design a Helmholtz resonator (HR) based acoustic absorber for low-frequency absorption. Baorui Pan^21^ developed a CNN-GA hybrid optimization framework that combines the advantages of traditional heuristic algorithms and emerging machine learning technologies to accelerate the inverse design of a random sound-absorbing structure with a diffusion field-based thickness of only 30 mm. Nansha Gao^22^ used the transmission matrix method to establish an acoustic sink model and created a database of 79,730 spectral lines within the frequency range of 0–5 kHz. The proposed CNN model, consisting of two building blocks (encoder and decoder), can predict the corresponding geometric parameters for the selected (expected) absorption curve.

In this design process, optimizing performance and lightweight structural design are particularly important. Jiang et al.^23^ optimized the acoustic performance of PU foam plastics using orthogonal methods. Xiong et al.^24^ achieved multi-objective lightweight design integration of the front-end structure of vehicles. Chen et al.^25^ studied multi-objective optimization of acoustic performance of polyurethane foam composite materials. Liu et al.^26^ investigated multi-objective optimization problems for low-frequency broadband microwave absorber metamaterial coding. Huang et al.^27^ proposed a multi-objective interval analysis method for evaluating and optimizing the vibro-acoustic comfort inside vehicles.

This research focuses on the use of deep learning methods to correlate material structural parameters with their acoustic properties. Begin by analyzing formulas to generate data samples, which will then be used to establish and train deep learning models. Different parameter networks are trained on the training set and evaluated on the testing set to select the network with the best generalization performance. This model could fit the underlying patterns between material structure and acoustic properties. The trained deep neural network could predict the sound absorption coefficient curve over a wide frequency spectrum of 0–5000 Hz in a single step. Secondly, the effectiveness of the network in acoustic reverse design is demonstrated through a typical design case. Finally, the MPP-SAC sound absorption material is optimized using the NSGA-II algorithm to achieve minimum material mass while maximizing the average sound absorption coefficient.

## Theoretical model

### The MPP-SAC composite structure model

**Fig. 1:**
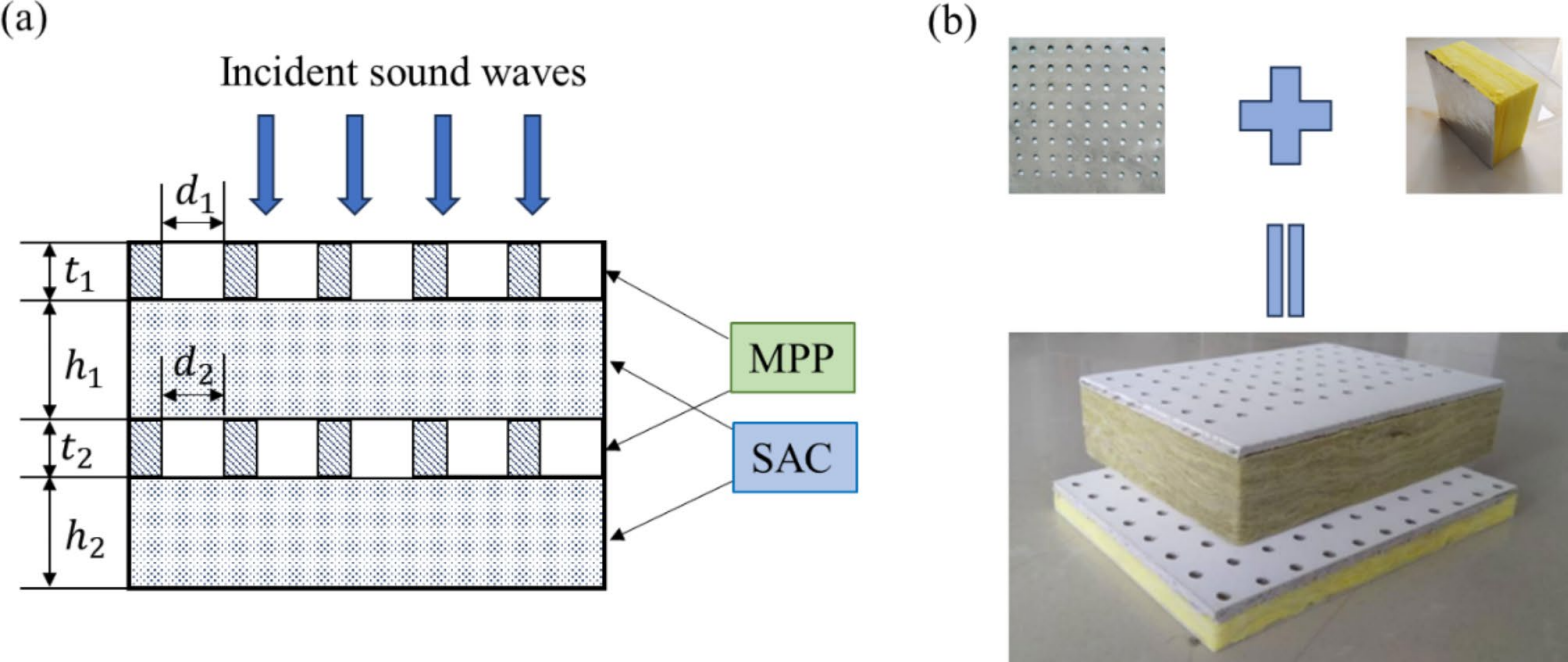
The double-layer MPP-SAC composite structure.

The schematic diagrams of the proposed composite structures are shown in Fig. 1a. The MPP is acoustical materials specially designed with a multitude of tiny apertures on its surface^28^. A layer of SAC material has been filled in the cavity beneath the structure of MPP, thus forming a single-layer MPP-SAC composite structure. However, to achieve efficient broadband sound absorption, increasing the number of layers to create a double-layer MPP-SAC composite material is the most direct approach. As shown in Fig. 1b, the double-layer structure is formed by stacking once on the single-layer foundation.

### Derivation of sound absorption formula

For MPP-SAC sound absorbers, at the normal incidence of sound wave the sound absorption coefficient α could be obtained by Maa et al.^5,6^

1 $$\alpha =\frac{{4\operatorname{Re} ({Z_c})}}{{{{[1+\operatorname{Re} ({Z_c})]}^2}+{{[\operatorname{Im} ({Z_c})]}^2}}}$$

where, ${Z_c}$ refers to the relative acoustic impedance of the MPP-SAC. Re() and Im() denote the real and imaginary part.

According to the sound absorption structure theory of MPP^5,6^, the acoustic impedance of MPP

2 $${Z_i}={R_i}+j\omega {M_i}$$

where, $j=\sqrt { - 1}$ is an imaginary unit, *i* is the number of layers, $\omega =2\pi f$, *f* is the frequency of the sound wave, ${R_i}$ is the acoustic resistance of the *i* layer of MPP, ${M_i}$ is the sound quality of the *i* layer of MPP.

The normalization of ${M_i}$ is carried out

3 $${z_i}={Z_i}/{Z_0}={r_{pi}}+j\omega {m_{pi}}$$

4 $${r_{pi}}=\frac{{32\eta {t_i}}}{{{\sigma _i}{\rho _0}{c_0}{d_i}^{2}}} \cdot \left[ {{{\left( {1+\frac{{{k_i}^{2}}}{{32}}} \right)}^{\frac{1}{2}}}+\frac{{\sqrt 2 }}{{32}} \cdot \frac{{{k_i}{d_i}}}{{{t_i}}}} \right]$$

5 $${m_{pi}}=\frac{{{t_i}}}{{{\sigma _i}{c_0}}} \cdot \left[ {1+{{\left( {9+\frac{{{k_i}^{2}}}{2}} \right)}^{ - \frac{1}{2}}}+0.85\frac{{{d_i}}}{{{t_i}}}} \right]$$

where${k_1}=\frac{{{d_1}}}{2}\sqrt {\frac{{\omega {\rho _0}}}{\eta }}$, and ${Z_0}={\rho _0}{c_0}$ is the characteristic impedance of air with ${\rho _0}=1.21\,\,{\text{kg/}}{{\text{m}}^3}$ and ${c_0}=343.4\,\,{\text{m/s}}$ being the density and sound speed of air, respectively. $\eta$ is the viscosity coefficient of air, usually $1.81 \times {10^{ - 5}}\,\,{\text{Ns/}}{{\text{m}}^2}$ at normal temperature and pressure. ${r_{pi}}$ is the relative acoustic resistance of the layer *i* MPP, ${m_{pi}}$ is the relative sound mass of the *i* layer MPP. ${t_i}$ (mm) is the thickness of the *i* layer of MPP, ${d_i}$ (mm) is the hole diameter of the *i* layer of MPP, ${\sigma _i}$ is the perforation rate of the *i* layer of MPP, and ${k_i}$ is the constant of the *i* layer of MPP.

Because the Delany–Bazley model only needs to know the flow resistance of SAC^9^, the model can determine the surface characteristic impedance and propagation constant of SAC, which greatly reduces the complexity of calculating the sound absorption coefficient. However, the model has a defect that the impedance calculated at low frequency is not positive real number. The D-B model is modified to Delany-Bazley-Miki model^10^. The surface acoustic impedance calculated by the new model is always a positive real number, and the accuracy is also high. The surface acoustic impedance of SAC could be derived by the improved D-B model^10^

6 $${Z_{si}}= - j \cdot {Z_{pi}} \cdot \cot ({K_{pi}} \cdot {h_i})$$

7 $${Z_{pi}}={\rho _0}{c_0}\left[ {1+5.501{{\left( {\frac{{1000 \cdot f}}{{{\delta _i}}}} \right)}^{ - 0.612}} - j8.431{{\left( {\frac{{1000 \cdot f}}{{{\delta _i}}}} \right)}^{ - 0.612}}} \right]$$

8 $${K_{p1}}=\frac{\omega }{{{c_0}}}\left[ {1+7.811{{\left( {\frac{{1000 \cdot f}}{{{\delta _1}}}} \right)}^{ - 0.618}} - j11.41{{\left( {\frac{{1000 \cdot f}}{{{\delta _1}}}} \right)}^{ - 0.618}}} \right]$$

where${Z_{pi}}$is the surface characteristic impedance of the *i* layer of SAC, ${K_{pi}}$ is the propagation constant of the *i* layer of SAC, ${h_i}$ (mm) is the thickness of the *i* layer of SAC, and ${\delta _i}$ is the flow resistance of the *i* layer of SAC.

Then, the impedance transfer method is used here to obtain the relative acoustic impedance of the structure in the following form

9 $${Z_c}=\left( {{z_1}+\left( {\frac{{{z_2} - j \cdot \cot ({h_2} \cdot {K_{p2}}) \cdot \cot ({h_1} \cdot {K_{p1}})+j}}{{{z_2} - j \cdot \cot ({h_2} \cdot {K_{p2}}) \cdot j+\cot ({h_1} \cdot {K_{p1}})}}} \right)} \right) \cdot {Z_p}/{Z_0}$$

To demonstrate the broad-band high sound absorption performance advantages of the double-layer MPP-SAC composite structure, a controlled variable method was employed with four comparative groups. The first group consisted of the upper part structure of the double-layer MPP-SAC configuration, while the second group involved the lower part structure of the same. The third group comprised a double-layer MPP structure without SAC material, that is, the composite structure of MPP and cavity (CAV) composite structure and the fourth group featured the complete double-layer MPP-SAC composite structure. The structural parameters of the four comparison groups are summarized in Table 1.

**Table 1:** Table of parameters for four different types of sound absorbing structures.

Code	${d_1}$	${t_1}$	σ_1_	${h_1}$	${D_1}$	${d_2}$	${t_2}$	σ_2_	${h_2}$	${D_2}$
Type 1	0.3	0.4	0.06	20	/	/	/	/	/	/
Type 2	0.3	0.4	0.06	30	/	/	/	/	/	/
Type 3	0.3	0.4	0.06	/	20	0.3	0.4	0.06	/	30
Type 4	0.3	0.4	0.06	20	/	0.3	0.4	0.06	30	/

**Fig. 2:**
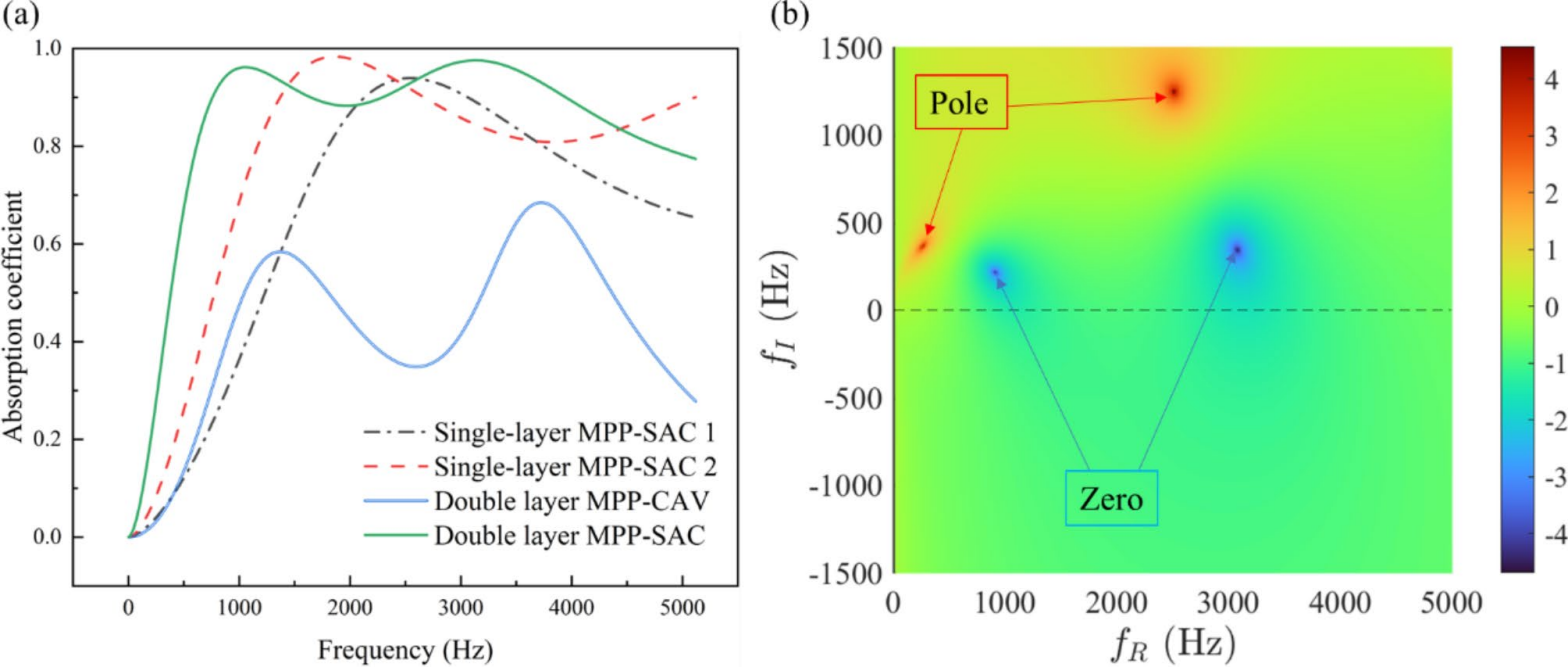
(**a**) Numerical absorption coefficient spectra of four groups of structures. (**b**) The complex frequency plane representation of the MPP-SAC absorber.

The parameters from the four groups in the table were individually input into the formula derived in the previous section to calculate the sound absorption coefficients. The spectral plots of sound absorption coefficients versus frequency were obtained, as shown in Fig. 2a. It is evident that the fourth group, the double-layer MPP-SAC composite structure, exhibits the highest sound absorption performance. To further investigate the acoustic absorption mechanism of the designed composite structure, graphical methods were employed to study the distribution of the reflection coefficient (R) within the complex frequency surface, as shown in Fig. 2b. Generally, under lossless conditions, the reflection coefficient includes conjugate zeros and poles^29^. Achieving perfect absorption at a specific frequency entails zeros precisely located on the real axis. The graph depicts the distribution of lg(|R|^2^) within the complex frequency surface for this composite acoustic structure. It reveals that near the real axis, where zeros are located, near-perfect absorption could be achieved at resonance frequencies. Conversely, when zeros are far from the real axis, perfect absorption is not achieved. The distance between zeros and poles also characterizes the bandwidth of acoustic absorption. This analysis provides insights into the superior acoustic performance of the double-layer MPP-SAC composite structure, particularly highlighting its wide-band high absorption capability.

## Deep learning model

### Generation of data sets

The dataset plays a crucial role in training and performance of deep learning models^11^. Effective training of neural networks relies heavily on sufficient data, and the volume directly impacts the model performance. A large dataset enhances the model’s generalization, leading to robustness and accuracy in real-world problem-solving scenarios. Therefore, data collection stands as a primary factor influencing the efficacy of neural network training. However, acquiring a substantial amount of data through experiments or simulations can be challenging. To quickly obtain a large-scale dataset, we applied the analytical formula from the previous section to generate data. Initially, the critical parameter ranges of the composite sound-absorbing material were determined. After analysis and verification, the primary parameter ranges for the composite structure were established as follows: micropore diameters ranging from 0.1 mm to 1 mm, thin plate thicknesses from 0.1 mm to 1 mm, perforation rates from 0.01 to 0.1, and SAC thicknesses from 1 mm to 100 mm. To ensure robust generalization capabilities of the neural network model post-training, a program was developed to randomly sample within the parameter space of each variable. In total, 60,000 data samples were generated. Among these, 80% were allocated to the training set and 20% to the test set^30^. This approach was adopted to swiftly acquire a substantial dataset for robust training and accurate evaluation of the neural network model performance.

Through the entire training approach of the neural network and the establishment of the dataset, it becomes evident that the structural parameter design of composite sound-absorbing materials is essentially a multi-input, multi-output regression problem. With the dataset developed above, the initial stage of neural network training has been successfully completed. With such a large-scale dataset, the well-constructed neural network can effectively utilize its advantages in fitting capability, thereby facilitating effective design of structural parameters for composite sound-absorbing materials.

### Data preprocessing

During the neural network training process, the features of structural parameter data exhibit varying scales. Direct computation using raw data may be susceptible to scale differences, complicating the model training process and hindering convergence. Normalization of the data aids in improving the efficiency and accuracy of model training by mitigating the impact of scale differences on model learning and prediction, thereby enhancing data interpretability^31^. In the forward network, the absorption coefficients at different frequency points share a uniform range (0, 1). However, different structural parameters span distinct value ranges. Therefore, for the inverse network tasked with predicting structural parameters, data preprocessing methods are essential. This study adopts the Min-Max normalization technique, which scales all raw input data to the range of (0, 1), as illustrated^32^:

10 $$x=\frac{{{x_0} - {x_{\hbox{min} }}}}{{{x_{\hbox{max} }} - {x_{\hbox{min} }}}}$$

This normalization approach ensures consistency in data handling and facilitates robust neural network training suitable for optimizing the design of composite sound-absorbing materials.

### Forward network model

Firstly, a function was defined to construct the forward neural network model. This function takes parameters including the input dimension, the number of units in the hidden layers, and the output dimension. Within the function, an input layer is first created, followed by the definition of hidden layers using dense layers. Given that the design of structural parameters for composite sound-absorbing materials involves nonlinear relationships, the rectified linear unit (ReLU) activation function is employed to enhance the network’s capacity for nonlinear fitting^33^. Subsequently, an output layer is defined using a linear activation function. The model function from TensorFlow is utilized to connect the input and output layers, thereby creating a complete model. The mean squared error (MSE) serves as the loss function, quantifying the discrepancy between predicted and actual values. A lower loss indicates better predictive performance and robustness of the model. The Adam optimizer is employed to dynamically adjust gradients during training, ensuring efficient convergence of the neural network. Before training commences, the weights and biases of each layer in the neural network are initialized randomly using a Truncated Normal distribution with a standard deviation of 0.1. To balance fitting accuracy and computational efficiency, a learning rate of 0.001 is set. During training, the stochastic gradient descent method is employed with mini-batches of size 32, dividing the dataset of 60,000 samples into smaller batches. With these parameters configured, the construction of the forward prediction network is complete. The subsequent steps involve loading the previously established dataset and utilizing Python programming within the Jupyter Notebook environment to train this dataset using the TensorFlow framework.

**Fig. 3:**
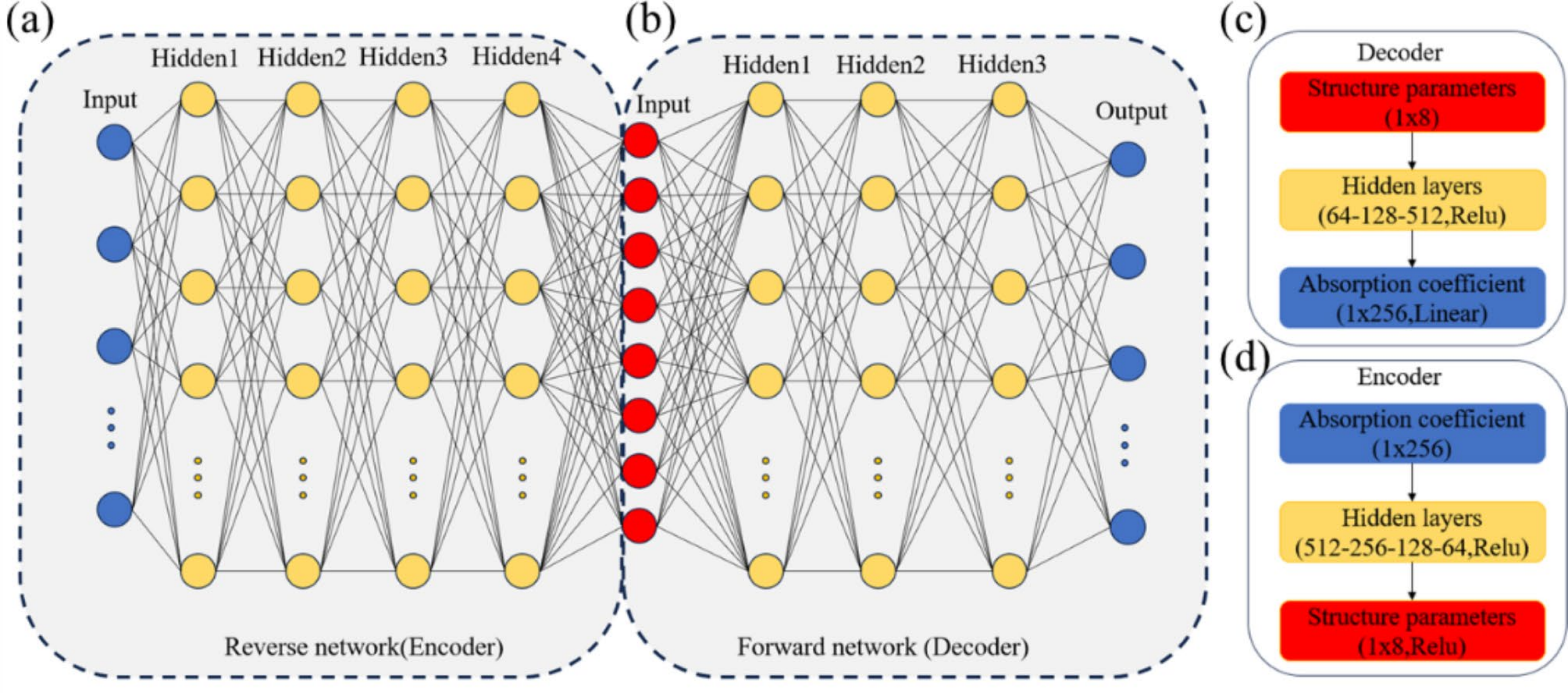
Deep neural network framework model. (**a**) Reverse network structure model. (**b**) Forward network structure model. (**c**) Network settings in the decoder. (**d**) Network settings in the encoder.

The forward prediction network maps structural parameters to absorption coefficient curves, achieved through a general fully connected neural network structure. To optimize fitting while minimizing computational complexity, the forward prediction network for composite structures comprises three hidden layers. The network architecture consists of four layers with neuron counts sequentially set as 64, 128, 512, and 256. The input layer has 8 neurons, as shown in Fig. 3b, c. Given that the absorption coefficient curve data, serving as output labels, is discretized into 256 points, the neural network at this layer has 256 neurons, each representing absorption coefficients at frequencies from 0 to 5120 Hz.

**Fig. 4:**
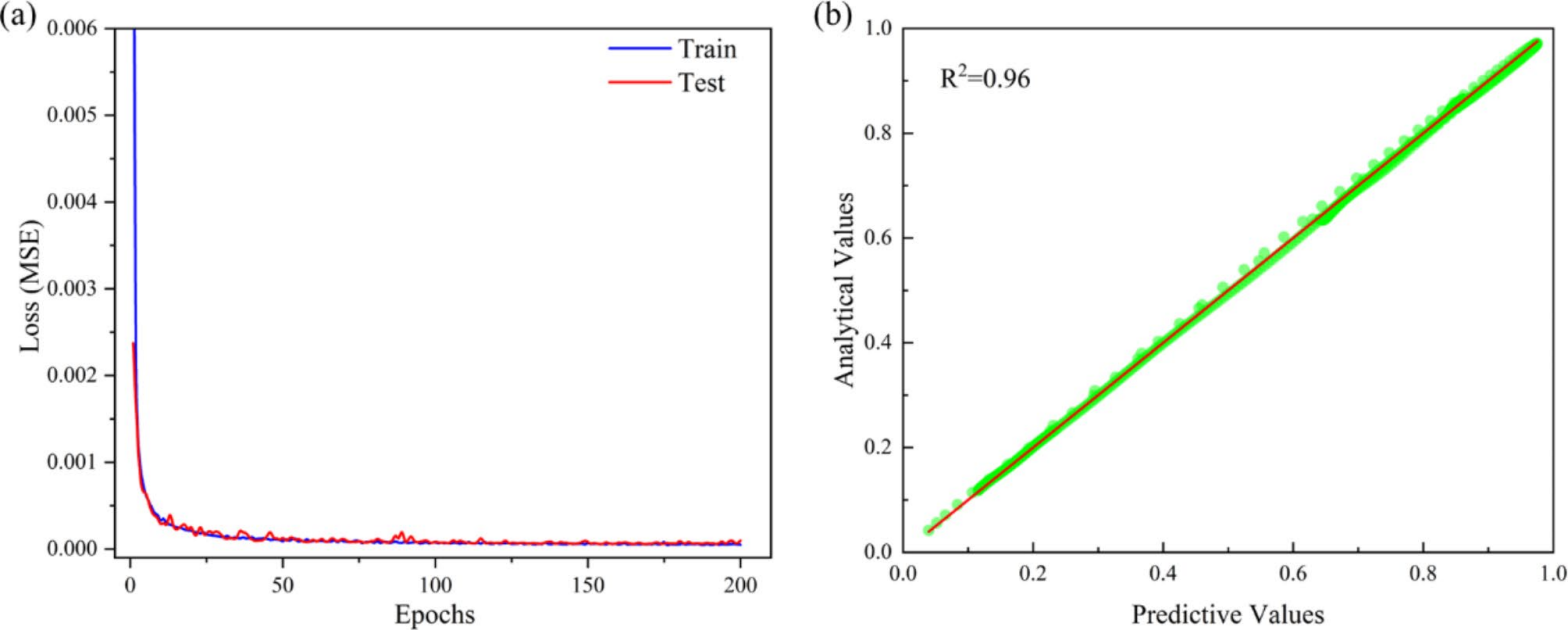
(**a**) The loss function on the training set and the test set in the forward network. (**b**) The R^2^ test between the predicted and analytical values in the forward network.

After 200 iterations, the feedforward prediction network achieved excellent convergence, with a final MSE less than 0.0001. As shown in Fig. 4a, the loss curves of the neural network on the training and test sets indicate rapid convergence and smooth curves, suggesting a smooth training process without significant fluctuations. Analysis of the average errors on the training and test sets yielded results of 0.445% and 0.476%, respectively. The small average errors imply minimal differences between predicted and actual values, confirming the accuracy of the deep learning model. Additionally, several randomly selected sets of absorption coefficient curves from the test set were subjected to an R-squared (R^2^) test, as depicted in Fig. 4b, resulting in a score of 0.96.

$${{\text{R}}^2}=1 - \frac{{S{S_{res}}}}{{S{S_{tot}}}}$$$$S{S_{res}}$$ is the sum of squared residuals, which is the sum of the squares of the differences between the predicted values and the actual values. $$S{S_{tot}}$$ is the total sum of squares, which is the sum of the squares of the differences between the actual values and their mean.

**Fig. 5:**
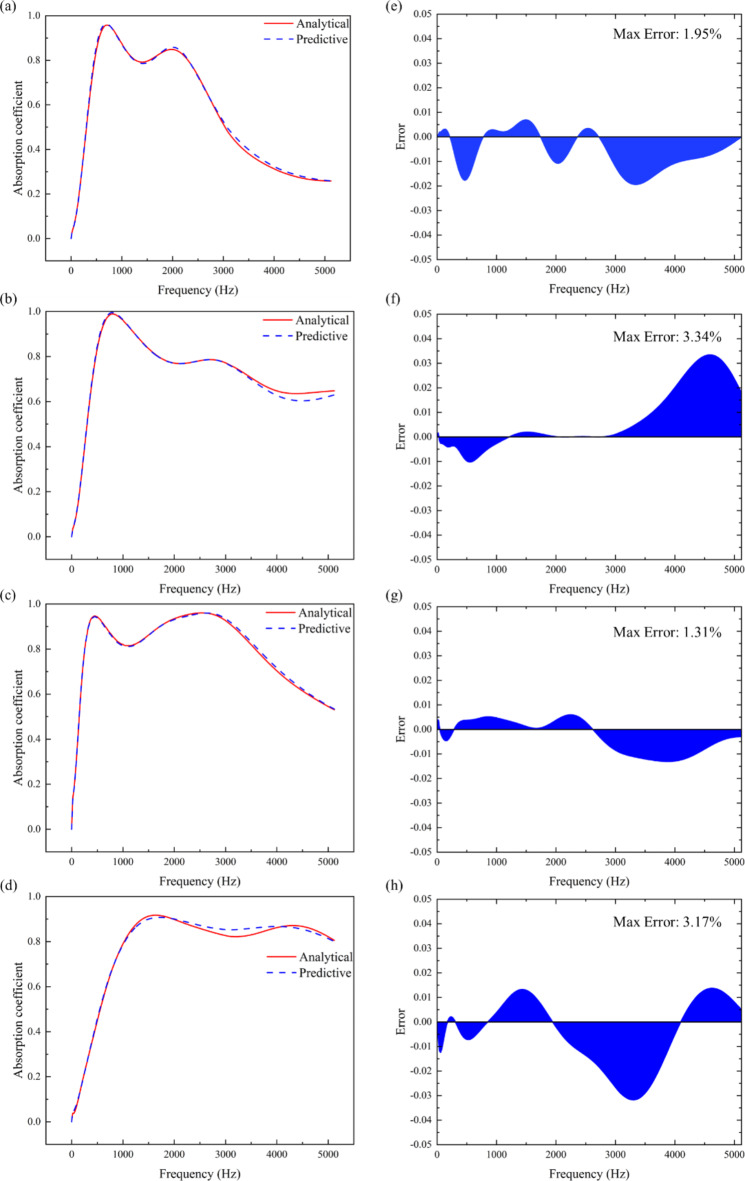
(**a**–**d**) The sound absorption coefficients predicted by the four groups of random parameters in the forward network are compared with the analyzed sound absorption coefficients. (**e**–**h**) The error between the predicted values and the analyzed values.

To validate the effectiveness of the forward prediction network, case studies are conducted to verify the accuracy of the neural network. Parameters for composite sound-absorbing materials are randomly set within the parameter space. Subsequently, this dataset is sequentially inputted into the forward prediction network. The predicted absorption coefficient curves generated by the neural network are then compared against those obtained through analytical solutions. The results are depicted in Fig. 5a–d.

The figure depicts four randomly selected instances where the neural network predicted typical scenarios under different parameters. It could be observed that the absorption coefficient curves predicted by the neural network exhibit similar characteristics to those obtained from analytical solutions in terms of the frequency, width, and magnitude of absorption peaks, showing a close alignment. Moreover, the error between the predicted and analytically derived absorption coefficients is calculated to be within 5%, as shown in Fig. 5e, h. From this, it could be concluded that the trained forward network possesses the capability to predict absorption coefficient curves. This network could potentially replace the process of calculating absorption coefficient curves based on structural parameters. Therefore, the decoder component of the deep learning model for structural parameter design of MPP-SAC has been successfully developed.

### Reverse network model

During the process of reverse engineering, a common issue arises known as “non-uniqueness,” where different sets of structural parameters exhibit similar characteristics^34^. This phenomenon often leads to oscillations during neural network training and affects the convergence of the cost function, thereby compromising the fitting performance of the neural network. Additionally, the structural parameters obtained from reverse engineering typically contain errors, further contributing to suboptimal performance.

To address the aforementioned issues, a deep learning model resembling an auto-encoder was constructed for structural parameter design^35^. The neural network was divided into two parts: a reverse network and a forward network. Reverse engineering is deemed more crucial and challenging than forward prediction. The architecture of the deep learning neural network model for reverse design is roughly opposite to that of the forward prediction network. MSE served as the loss function, and Adam optimization algorithm was employed to dynamically adjust the model gradients. Training the reverse design network proves challenging as it needs to integrate with the forward prediction network, which could impact the training of the reverse network to some extent. Therefore, expanding the depth and width of the network was implemented to ensure effective training. In the structural parameter design, the reverse design network comprises four hidden layers. To balance fitting accuracy and training speed, the number of neurons in each layer was set as follows: 512 neurons in the first layer, 256 in the second, 128 in the third, and 64 in the fourth, with 256 neurons in the input layer, as depicted in Fig. 3a, d.

**Fig. 6:**
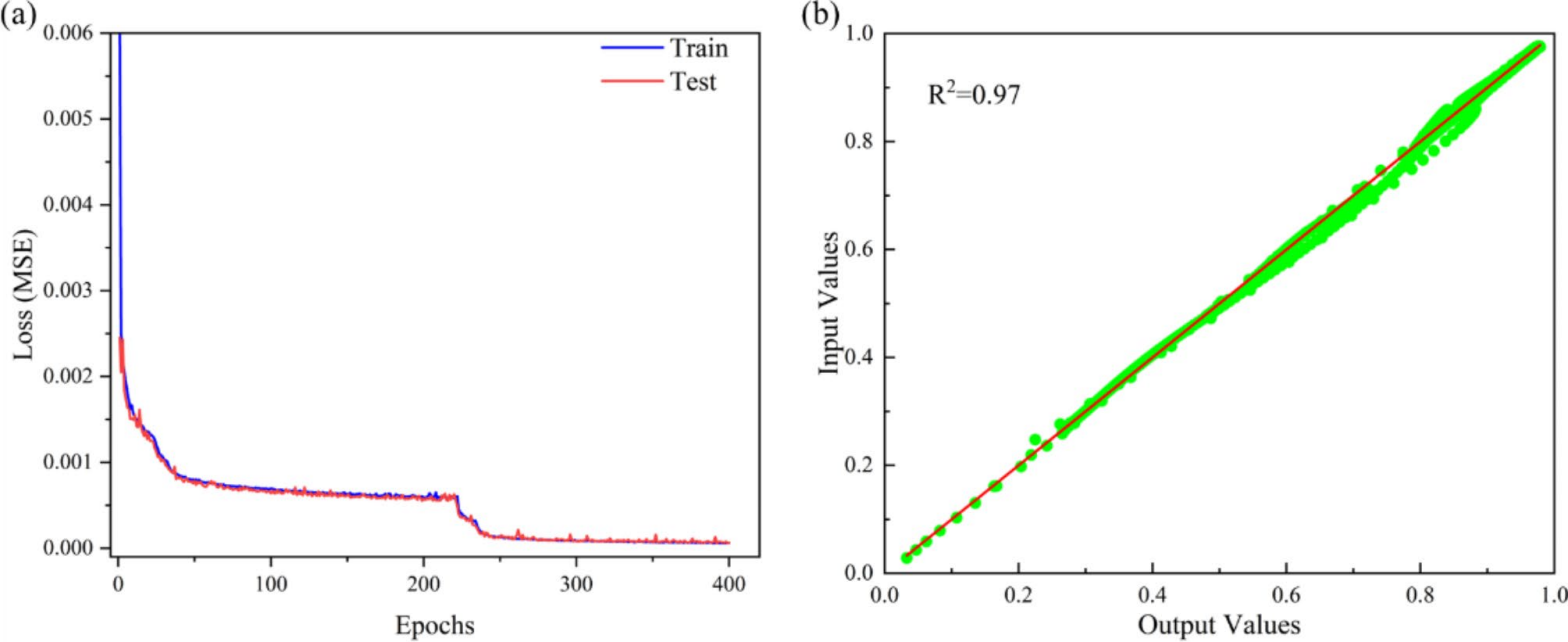
(**a**) The loss function on the training and test sets in the reverse network. (**b**) The R^2^ test between the output and input values in the reverse network.

After 400 iterations of training, the loss function curves on the training and test sets are depicted in Fig. 6a. A favorable convergence was achieved around 300 iterations, with a final MSE of less than 0.0001. Analysis of the average errors on the training and test sets yielded results of 0.445% and 0.476%, respectively. To further validate the model’s accuracy, several sets of random samples from the entire test set were chosen to evaluate the absorption coefficient curves using an R^2^ test, as shown in Fig. 6b. The final R^2^ value obtained was 0.97.

**Fig. 7:**
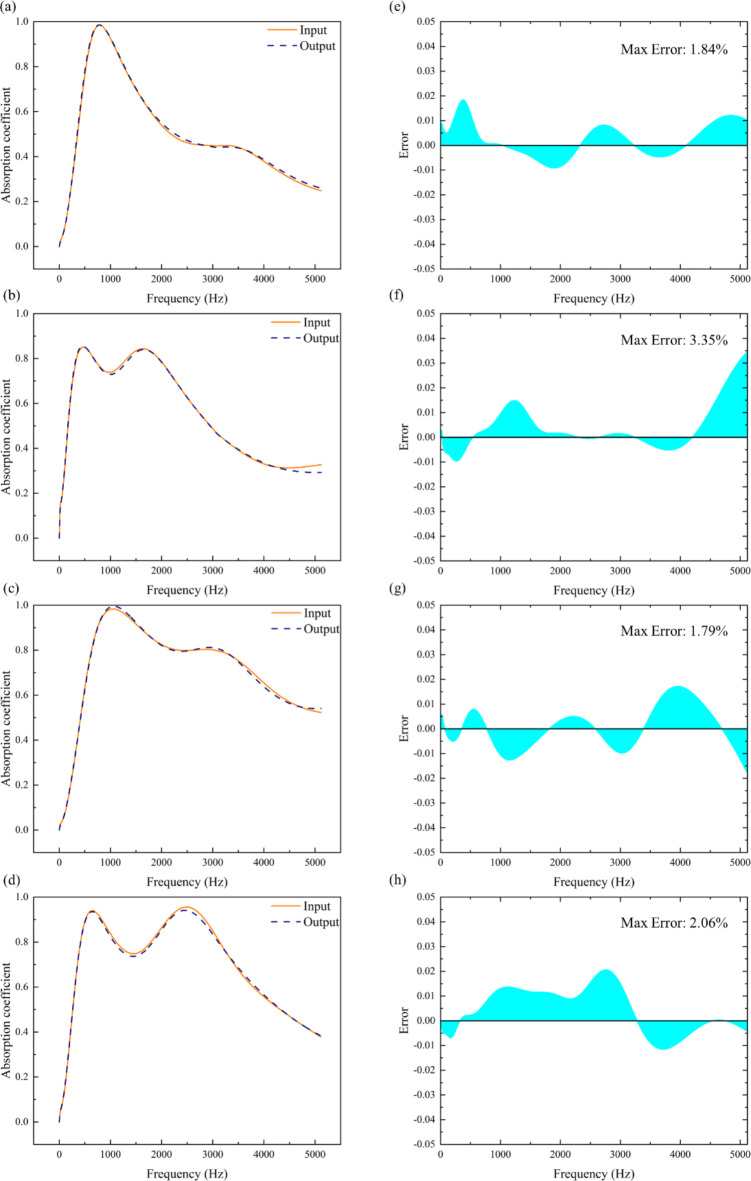
(**a**–**d**) The sound absorption coefficients output in the reverse network are compared with the input sound absorption coefficients. (**e**–**h**) The error between the output values and the input values in the reverse network.

To validate the trained class of autoencoder deep learning model’s ability to encode and reconstruct input absorption coefficient curves, several randomly selected curves from the test set were fed into the neural network. The resulting output curves were then compared with the input curves, as depicted in Fig. 7a–d. From the figures, it is evident that within the frequency range of 5120 Hz, the input curves retain their original features after processing through the neural network. The output results closely match the original curves, demonstrating a high level of consistency. By calculating the error between the input and output absorption coefficients, it was found that the errors are all below 5%, as shown in Fig. 7e–h. Therefore, it could be concluded that the neural network obtained after training has acquired the capability for inverse design. Consequently, the entire deep learning model has now been fully completed.

## Broadband high sound absorption performance of MPP-SAC case design

**Fig. 8:**
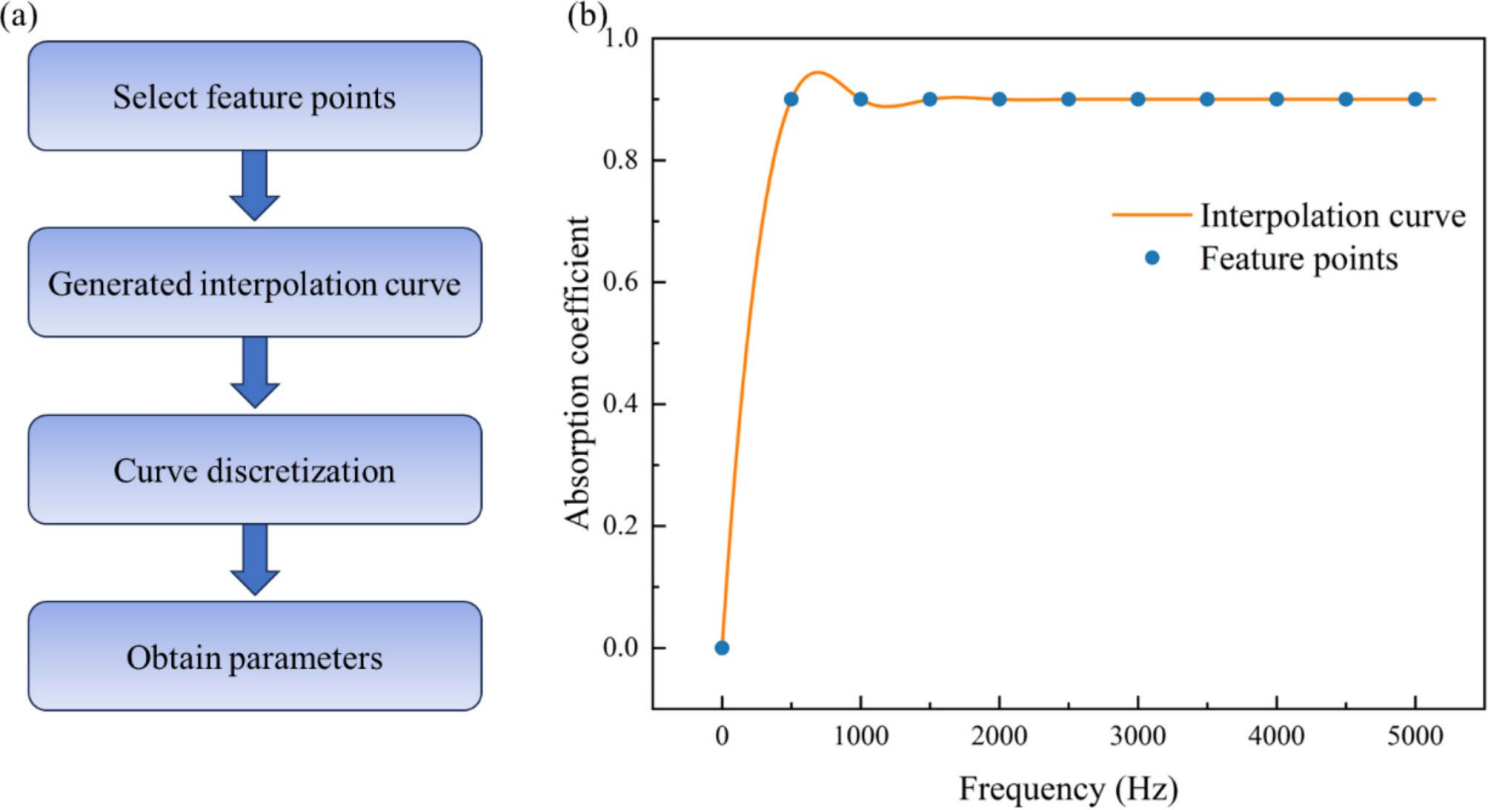
(**a**) Get a flowchart of the target curve. (**b**) Quadratic spline interpolation fits the curve.

In this section, a pre-trained neural network is employed to design the optimal structural parameters of acoustical materials with broadband high sound absorption performance. Since the entire deep learning model takes as input 256 discrete points from the sound absorption coefficient curve, the first step is to generate the target curve. To obtain the desired sound absorption coefficient curve conveniently. Firstly, select key points and fit the target absorption curve using quadratic spline interpolation. Subsequently, discretize the absorption coefficient values of this curve at every 20 Hz interval to obtain 256 sample points. Finally, these points are normalized and used as input labels fed into the pre-trained deep learning model, as shown in Fig. 8a,b. The model quickly provides design results at the intermediate layer, with the following parameters:${d_1}=0.29{\text{mm}}$, ${t_1}=0.41{\text{mm}}$, ${\sigma _1}=0.0067$, ${h_1}=10.7{\text{mm}}$, ${d_2}=0.31{\text{mm}}$, ${t_2}=0.10{\text{mm}}$, ${\sigma _2}=0.0068$, ${h_2}=67.4{\text{mm}}$.

**Fig. 9:**
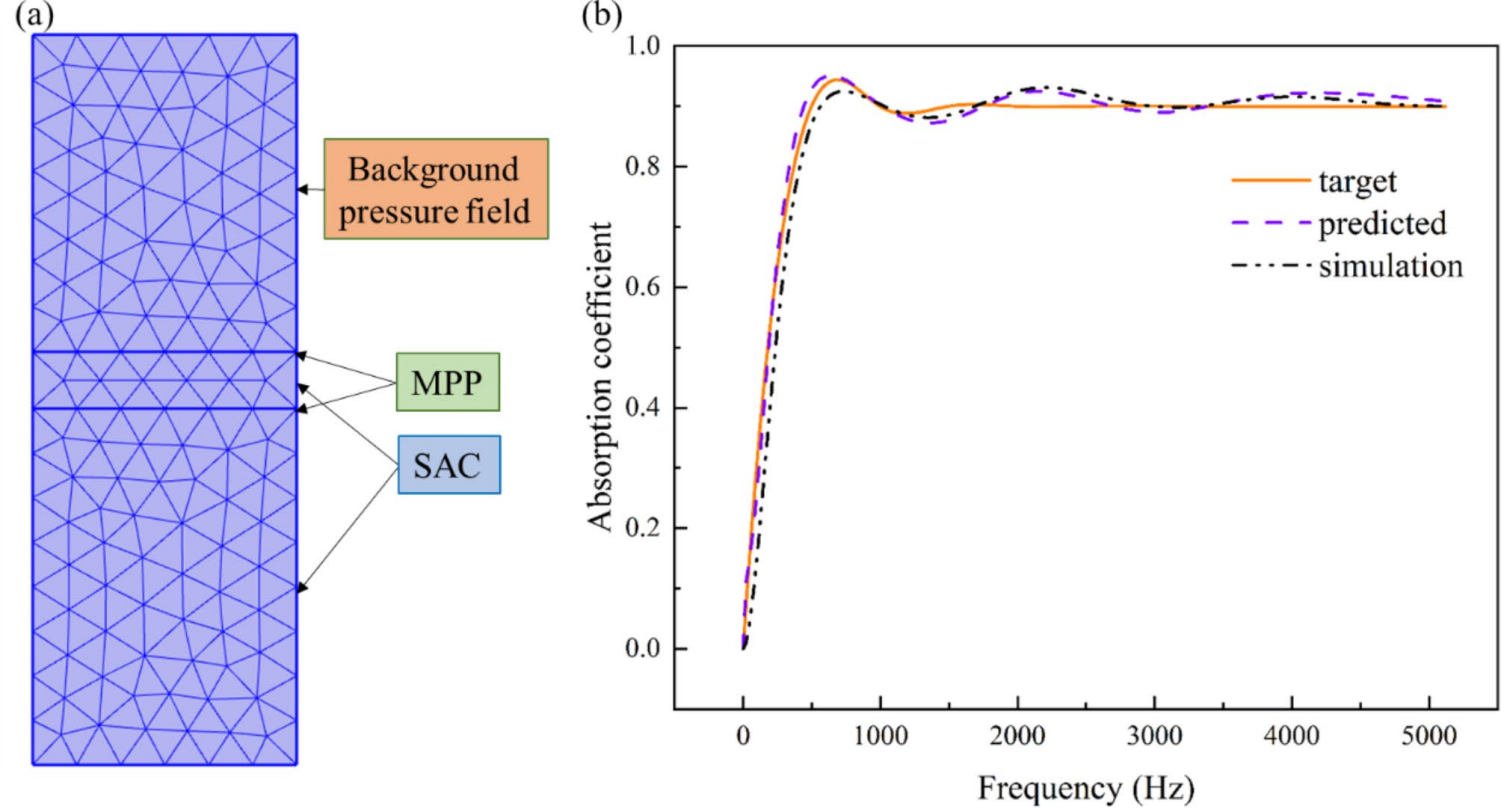
(**a**) Simulation of MPP-SAC in COMSOL Multiphysics. (**b**) The difference between the target absorption coefficient curve and the prediction and simulation.

To further validate the structural parameters designed by the deep learning model, conducted finite element simulation analysis of the sound absorption coefficient using the commercial finite element software COMSOL Multiphysics, as shown in Fig. 9a. The acoustical model employing the Delany-Bazley-Miki approach for porous media is illustrated. The upper portion of the figure shows the interaction region of the incident pressure field, where the incident wave is a plane wave with an amplitude of 1. The lower section depicts the MPP-SAC composite structure, with the solid line in the middle representing the internal perforated plate configuration. The solid line at the bottom signifies a hard acoustic boundary, while the periodic boundaries on either side of the structure denote an infinite extent. The simulation results, represented by the black dashed line in Fig. 9b, show that the predicted absorption coefficient curve closely matches the simulated curve in terms of absorption peaks, widths, and frequencies. Additionally, both curves exhibit similar shapes and trends.

## Multi-objective optimization of MPP-SAC

The essence of multi-objective optimization problems lies in finding the optimal solutions for multiple objectives under variable constraints^36^. To enhance the sound absorption performance of MPP-SAC while simultaneously reducing its mass.

Because the genetic algorithm with multiple objective functions could also optimize only the objective function in the same direction, it could only take the maximum or minimum value simultaneously^37^, the optimization considers the negative of the average absorption coefficient. The absorption coefficient is defined as the ratio of absorbed sound energy to incident sound energy. However, the absorption coefficient varies with frequency. Therefore, the average absorption coefficient is widely used in engineering practice to assess sound absorption capability. The average absorption coefficient in this study is computed over the 0–5120 Hz frequency range using Formula (11). The mass per unit area (cm^2^) of the material is calculated based on its density and thickness, as described in Formula (12).

In summary, the MPP-SAC design multi-objective optimization model is obtained:

11 $${y_1}= - {\alpha _{{\text{average}}}}= - \frac{{\int_{0}^{{5120}} {\alpha df} }}{{5120}}$$

12 $${y_2}={M_{total}}={M_{MMP}}+{M_{SAC}}=[{\rho _{MMP}} \cdot ({t_1}+{t_2})+{\rho _{SAC}} \cdot ({h_1}+{h_2})] \cdot S$$

$$Find:X=({X_{t1}},{X_{t2}},{X_{h1}},{X_{h2}})$$

$$Min:Y=({y_1},{y_2})$$

$$Subject$$ $$to:({t_1},{t_2} \subseteq (0.1\,\,{\text{mm}},1\,\,{\text{mm}});{h_1},{h_2} \subseteq (10\,\,{\text{mm}},\,\,100\,\,{\text{mm}}))$$

${\rho _{{\text{MPP}}}}$ in the formula is 1000 kg/m^3^, ${\rho _{{\text{SAC}}}}$ in the formula is 80 kg/m^3^.

**Fig. 10:**
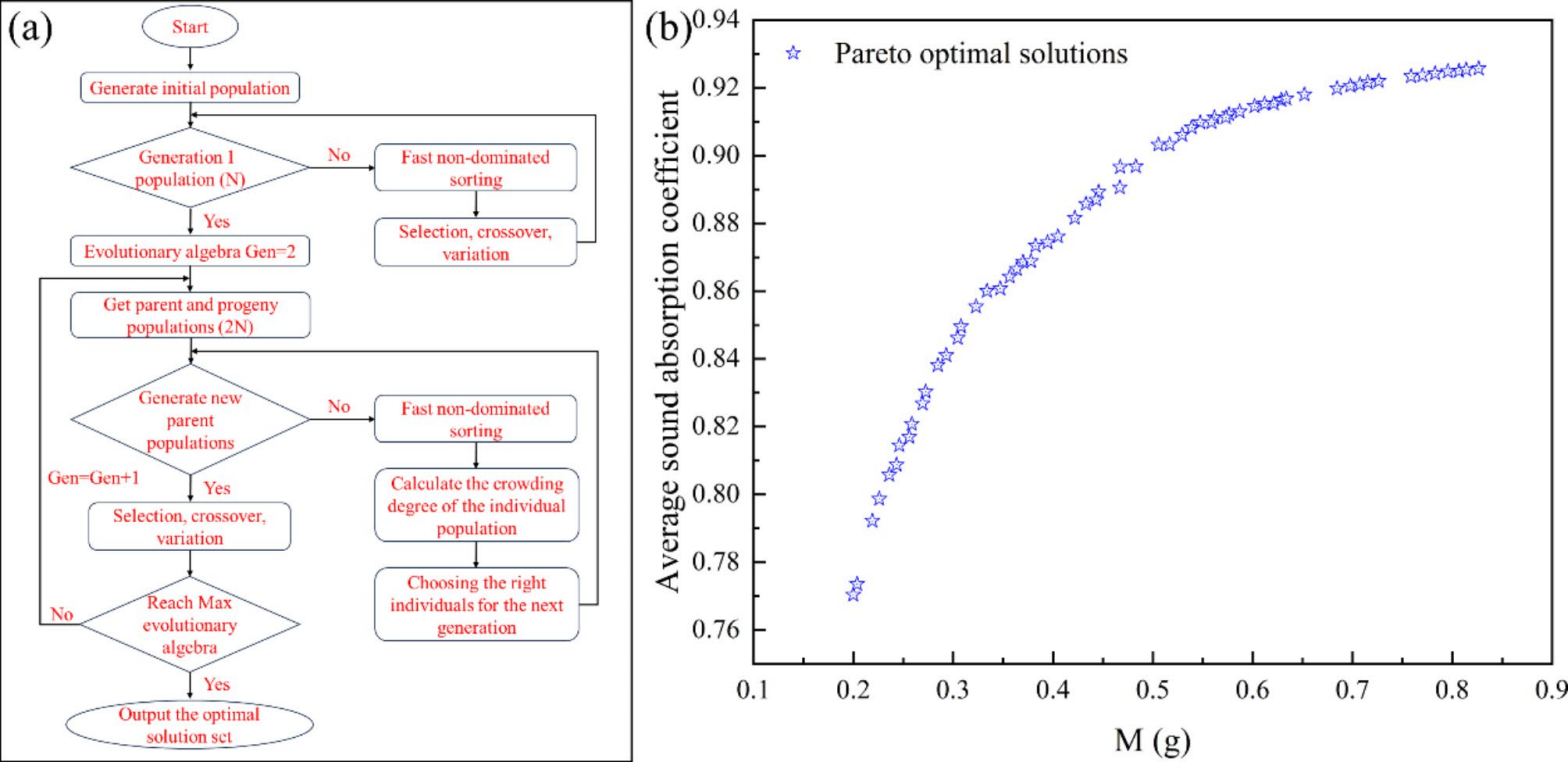
(**a**) Flow diagram of the NSGA-II algorithm. (**b**) The Pareto optimal solution set.

To solve the dual-objective constrained optimization problem considering average absorption coefficient and total mass, obtained the Pareto optimal solution set using the Non-dominated Sorting Genetic Algorithm II (NSGA-II). NSGA-II is one of the most widely applied algorithms for multi-objective optimization due to its ability to reduce the complexity of non-dominated sorting genetic algorithms. It is known for its fast execution speed and good convergence of solution sets. The NSGA-II is an algorithm that simulates the evolutionary mechanism of organisms in nature, following the law of survival of the fittest. In scientific or production practice, it enables us to find the solution that best meets the requirements of the environment among all possible solutions^38^. The basic flowchart of NSGA-II is illustrated in Fig. 10a. The NSGA-II first creates a random initial population and a series of new populations from the previous generation. It stops when one of the stopping criteria (maximum number of generations, maximum computation time, tolerance to the objective function) is satisfied. In the NSGA-II, the population is first initialized, the fitness function of individuals is calculated, and then the fitness function of individuals is scored by evolutionary operations such as selection, crossover, and mutation and by evaluating the fitness value score of individuals, those with higher scores are evolved to the next generation, at the same time, those with lower scores are eliminated^38^. By continuously following the direction set by the fitness function, the next generation’s population inherits the more adapted information from the population of the previous generation, so the parameters being optimized during evolution keep approaching the direction set by the objective function^39^. In NSGA-II, solutions are sorted based on non-domination and crowding distance to maintain the non-dominance concept and promote diversity in the solution set. For better convergence and faster calculations, set the population size and the maximum number of iterations to 200 and 500, respectively. The population size is set to 200, which is a commonly used value that offers a balanced trade-off between solution exploration and computational cost. This relatively large population size enhances the ability to explore the solution space more thoroughly. The maximum number of iterations is set to 500, providing sufficient iterations for the algorithm to converge to a solution that is close to the optimal. This configuration enhances the algorithm’s performance in efficiently exploring the solution space while ensuring robustness in finding Pareto optimal solutions.

**Fig. 11:**
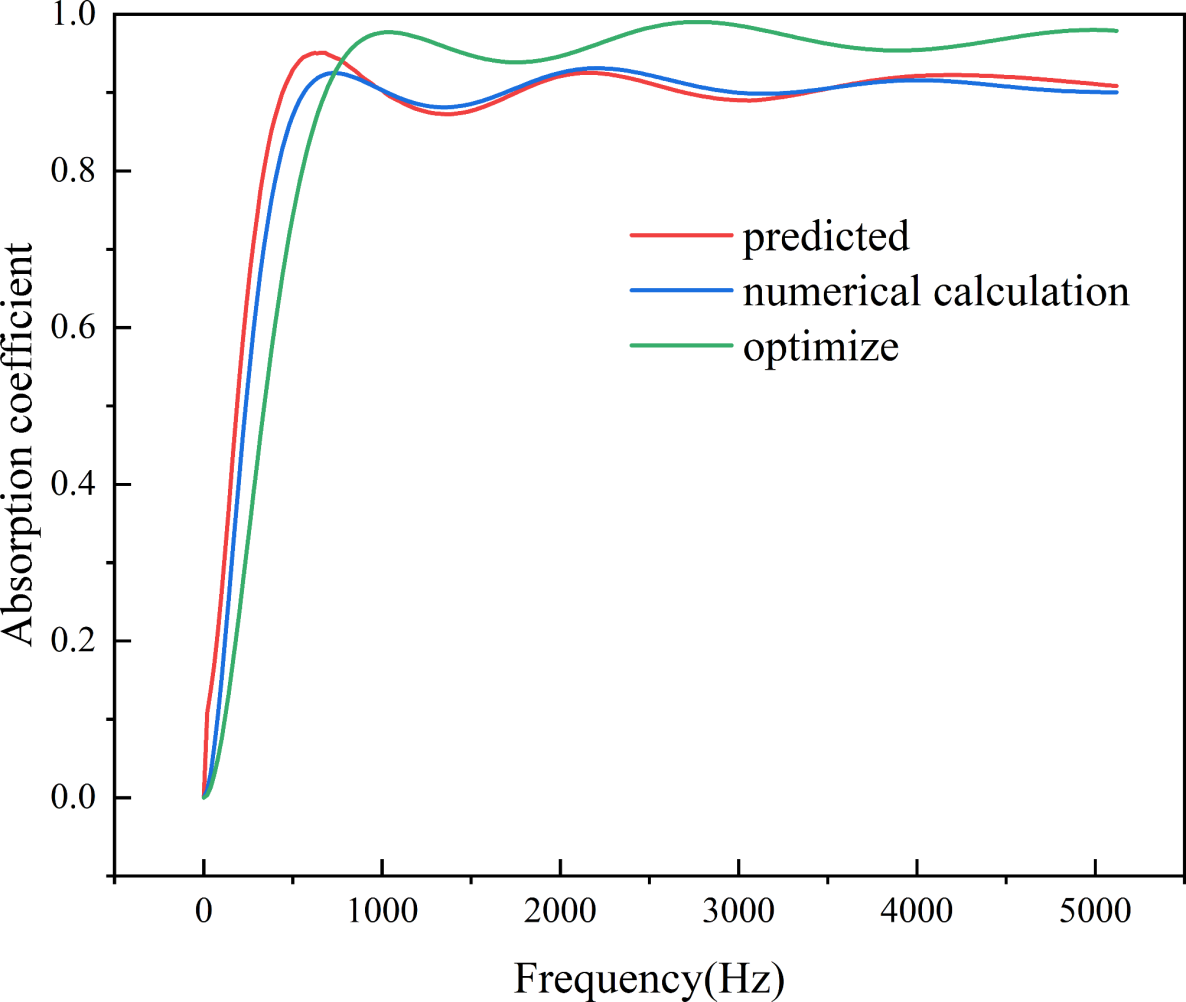
Comparison of the sound absorption coefficient of the optimized structure with the original structure.

The fundamental distinction between multi-objective optimization and single-objective optimization lies in the fact that optimizing one objective may decrease the performance of another, and optimizing all objectives simultaneously is nearly impossible; thus, it necessitates the use of weight allocation to achieve the best compromise across all objectives. Furthermore, the optimal solutions for multi-objective optimization problems often form a set of solutions rather than a single solution. Through 500 optimization computations, the Pareto optimal solution set was obtained, as shown in Fig. 10b. From the graph, it is observed that as material quality increases, the average absorption coefficient tends to increase. Simultaneously optimizing both objectives with equal weights aims to achieve a comprehensive optimal solution. In practical engineering applications, decision-makers could assign different weights to each objective based on requirements to select the optimal design solution. The structural parameters reversely designed in the previous section are brought into formula (11) and (12), the average sound absorption coefficient is 0.868 and the total mass is 0.6758 g. From the optimal solution set, 10 sets of solutions were selected where the average absorption coefficient is greater than 0.868 and the mass is less than 0.6758 g. As shown in Table 2, the average absorption coefficient and total mass were normalized, and a 1:1 weighting was applied to achieve a balanced comprehensive optimal solution. The comprehensive optimal solution obtained by the fifth set of structural parameters in the table is shown in Fig. 11, which shows the comparison between the absorption coefficient before optimization and that after optimization. The average sound absorption coefficient of 0.910 is increased by 4.84%, and the total mass is decreased by 18.98% to 0.5475 g.

**Table 2:** Optimized parameters for 10 sets of layered structures.

	Layers	h (mm)	t (mm)	d (mm)	$\sigma$	M (g)	${\alpha _{{\text{average}}}}$
1	Layer-1	43.79	0.150	0.384	0.095	0.5055	0.903
	Layer-2	15.45	0.166	0.459	0.045		
2	Layer-1	41.34	0.153	0.386	0.093	0.5168	0.903
	Layer-2	19.44	0.152	0.439	0.051		
3	Layer-1	44.62	0.152	0.384	0.095	0.5294	0.906
	Layer-2	17.14	0.201	0.429	0.050		
4	Layer-1	46.71	0.151	0.398	0.097	0.5388	0.908
	Layer-2	16.89	0.149	0.515	0.055		
5	Layer-1	47.66	0.150	0.370	0.098	0.5475	0.910
	Layer-2	17.08	0.146	0.619	0.054		
6	Layer-1	44.13	0.153	0.390	0.098	0.5588	0.910
	Layer-2	21.57	0.179	0.333	0.059		
7	Layer-1	47.76	0.148	0.374	0.098	0.5618	0.911
	Layer-2	18.74	0.150	0.664	0.068		
8	Layer-1	45.83	0.155	0.380	0.097	0.5731	0.911
	Layer-2	21.72	0.171	0.610	0.076		
9	Layer-1	47.73	0.149	0.384	0.097	0.5762	0.912
	Layer-2	20.45	0.159	0.600	0.070		
10	Layer-1	45.07	0.149	0.369	0.098	0.5870	0.912
	Layer-2	24.25	0.175	0.599	0.070		

## Conclusion and discussion

This study proposes a broadband sound absorption structure composed of MPP-SAC. A deep neural network approach based on deep learning autoencoders is introduced for the inverse design of geometric parameters of the sound absorption structure. The forward network achieves spectral prediction of structural parameters, while the inverse network predicts the geometric parameters of the absorber based on the input target spectrum. A sound absorption structure was designed, featuring a flat and wideband absorption plateau (α ≥ 0.9). The designed absorber was tested through simulation, and the results matched the numerical predictions. This technique has been successfully applied to existing simple dual-layer MPP-SAC absorbers and may be extended to complex metamaterial structures in the future. Utilizing deep learning for the inverse design of metamaterial structural parameters could achieve more accurate parameters, saving design time and increasing efficiency compared to traditional methods.

Subsequently, the NSGA-II algorithm was used to perform multi-objective optimization on the acoustic package material quality and the average sound absorption coefficient. After optimization, the average sound absorption coefficient of the acoustic package was 0.910, an improvement of 4.84%, and the total mass was reduced by 18.98% to 0.5475 g, ensuring sound absorption performance while achieving lightweight.

The application of deep learning methods in the design of acoustic absorption structures offers novel solutions to the inverse design problem of absorptive materials and provides a technological pathway from requirement capture, targeted design, to product implementation. This approach is advantageous for addressing vibration and noise issues in engineering through advanced technologies. Although inverse design of structural parameters for various scenarios can be achieved, the overall design remains less intelligent. Therefore, developing a unified deep learning model that can automatically select the acoustic absorption structure based on desired performance and provide corresponding design parameters represents a deeper and more meaningful research objective. This advancement would also foster the development of deep learning and artificial neural network technologies at the application level. It could offer more effective solutions for broadband acoustic absorption in engineering applications and promote the broader adoption of absorptive structures. However, in the field of acoustic metamaterials, numerous topological optimization problems persist, which can also be addressed through deep learning methods for inverse design based on target physical characteristics. Consequently, improving and extending such deep learning-based design methods to other structural design problems will become a prominent research direction in the future.

## Data Availability

The data that support the findings of this study are available from the corresponding author upon reasonable request.
